# Determination of perceived stress, salivary cortisol and interleukin-1β levels and clinical features of chronic urticaria in patients with dermographic urticaria in comparison to patients with chronic spontaneous urticaria and healthy individuals

**DOI:** 10.3389/fimmu.2026.1741412

**Published:** 2026-02-12

**Authors:** Liborija Lugović-Mihić, Ina Novak-Hlebar, Dajana Smoljan, Ines Vukasović, Ema Barac, Maja Vilibić

**Affiliations:** 1 Department of Dermatovenereology, University Hospital Center Sestre Milosrdnice, Zagreb, Croatia; 2 School of Dental Medicine, University of Zagreb, Zagreb, Croatia; 3 Department of Clinical Chemistry, University Hospital Center Sestre Milosrdnice, Zagreb, Croatia; 4 School of Medicine, Catholic University of Croatia, Zagreb, Croatia; 5 Dom zdravlja Zagreb, Family Physician Office, Zagreb, Croatia; 6 Department of Psychiatry, University Hospital Center Sestre Milosrdnice, Zagreb, Croatia

**Keywords:** chronic urticaria, comorbidity, cortisol, demographic urticaria, IL-1, inducible urticaria, saliva, stress

## Abstract

**Introduction:**

Although dermographic urticaria or symptomatic dermographism (SD), is the most common chronic inducible urticaria subtype, little is known about its etiopathogenesis and associated factors such as psychological stress.

**Methods:**

In this cross-sectional study we included a total of 100 examinees, and compared the data/findings of 34 SD/DU patients to 33 chronic spontaneous urticaria (CSU) patients, and 33 healthy controls. We looked at perceived psychological stress (Perceived Stress Scale/PSS), salivary cortisol and IL-1β levels, clinical characteristics [dermographism activity/severity (on a scale from 1 to 4), disease control (Urticaria Control Test/UCT) and disease activity of CSU patients (Urticaria Activity Score/UAS)], and demographic data.

**Results:**

Dermographism activity/severity linearly correlated with perceived stress/PSS (r=0.347; p=0.045), with no correlation with other factors. Disease duration in SD/DU patients was negatively linearly related to salivary cortisol (*r=-0.369; p=0.032*), but not to IL-1β. In the SD/DU and CSU groups, no significant correlations were observed between salivary cortisol, IL-1β, PSS, and age; however, urticaria severity positively, linearly correlated with perceived stress (*p=0.004*). Males had higher salivary cortisol (median 1.1 vs. 0.9; *p=0.028*) and higher IL-1β levels than females (687.6 vs. 408.9 pg/mL; *p=0.029*), while females had higher perceived stress levels (16 vs. 12; *p=0.006*). CSU patients had significantly higher disease control than the SD/DU group (moderate effect size) (*p=0.001*). In SD/DU patients with concomitant allergies, lower cortisol values were recorded (moderate effect size) (median log 1.03 vs. 0.80 nmol/L; p=0.032) and disease lasted longer than in those with no allergies (a large effect size) (48 vs. 18 months; p=0.002). In SD/DU patients with associated conditions/diseases, their SD/DU lasted significantly longer than for those without comorbidities (60 vs. 24 months; p=0.017).

**Conclusion:**

Since stress is often perceived by SD/DU patients and affects SD/DU activity/severity, psychological support could be beneficial for these patients.

## 1 Introduction

Among the frequent forms of inducible urticaria, dermographic urticaria (DU) [also known as symptomatic dermographism (SD) or urticaria factitia] is the most common (1–4). Dermographism is an exaggerated wheal and flare response that appears quickly, about 15–30 minutes after stroking, scratching, or pressing the skin, and can occur on various body parts (5–7). Whereas simple dermographism—where wheals usually appear within 6–7 minutes, without itch, and begin to fade 15–30 minutes later SD/DU occurs with itchy hives that appear within 5 minutes and usually last about 30 minutes, with itching becoming worse at night, with no stimulus, no urticaria and no sleep disturbance (5). Consequently, it often significantly impacts quality of life, particularly fatigue. Dermographism may appear alone or together with other skin conditions: atopic dermatitis, chronic spontaneous urticaria (CSU), and other inducible urticarias. Although onset is most common in young adults, it may appear at any age. According to some data, dermographism affects about 2–5% of the general population, but only some develop symptoms. Some studies have reported a positive family history (8, 9). Concerning sex and race, there are no extensive epidemiological studies to date. The exact etiopathogenesis of SD/DU is unclear but is known to involve the release of vasoactive mediators from skin mast cells, made evident by elevated serum histamine levels (5). SD/DU can have its onset after infections (bacterial, fungal, or viral) or allergy to drugs (e.g., penicillin), and may become worse with exercise, emotion, heat, cold, or stress. Also, skin trauma may trigger the release of an antigen that reacts with membrane-bound IgE on mast cells, leading to the release of inflammatory mediators (e.g., histamine). This is supported by data showing that dermographism can be passively transferred to others (9). SD/DU is a clinical diagnosis, elicited by firmly stroking the skin (usually across the back or forearm) and observing the reaction after several minutes for confirmation. A dermographometer or special testing device (namely the Fric Test – a plastic device with pegs of varying lengths) can be used to perform the challenge test by applying a range of pressures to the skin. This has to be performed after discontinuation of antihistamines (AHs), as they may cause false negative result (5, 10, 11). Simple dermographism requires no specific treatment, only the avoidance of triggers by wearing loose-fitting clothing, avoidance of irritants, and sometimes psychological counseling, anxiolytics, or antidepressants. For pharmacologic treatment of SD/DU, non-sedating second-generation H1-AHs (e.g., cetirizine, loratadine, desloratadine, bilastine, etc.), alone or in combination with H2-AHs, are used, potentially in high doses long-term. Other therapeutic options include montelukast, phototherapy, or omalizumab (5). (Delayed dermographism appears to be treatment-resistant) Dermographism may last for months, years, or even decades.

Among the potential triggers or etiological factors for SD/DU, psychological stress has frequently been mentioned in the literature. Psychological stress is defined as a psychological state in which an individual perceives that situational demands exceed their personal resources, involving a synchronized activation of psychological factors, as well as the neuroendocrine and immune systems (12). According to the psychoneuroimmunological model, a stressful event triggers negative emotions accompanied by physiological changes in the organism. These responses are mainly regulated by two components of the neuroendocrine system: the sympatho-adrenal-medullary (SAM) and the hypothalamic-pituitary-adrenal (HPA) axes. When an individual perceives stress, the hypothalamus releases corticotropin-releasing factor (CRF), which then stimulates the anterior pituitary gland to secrete adrenocorticotropic hormone (ACTH), subsequently inducing the adrenal cortex to produce cortisol (13, 14). Several immunological factors/biomarkers have shown associations with CU, and studies have demonstrated elevated levels of numerous proinflammatory cytokines in most chronic urticaria patients, including IL-1β, IL-6, tumor necrosis factor (TNF)-α, IL-2, IL-12, IL-17, IL-23, IL-13, and IL-18 (15–17). Among them, IL-1 plays a central role in the host defense response to injury: IL-1 directly mediates vascular and inflammatory responses and promotes vascular leakage (17). Thus, after mechanical trauma, IL-1β mediates plasma extravasation and recruitment of inflammatory cells to the skin, which enhances the local inflammatory response with consequent mast cell activation and hives, e.g. in SD/DU. The use of salivary biomarkers in diagnostics has become increasingly attractive due to its non-invasive nature and simplicity, which is particularly valuable for SD/DU patients with sensitive skin (18–23). For the analysis of salivary biomarkers, it is important to use standardized salivary tests such as those for IL-1β and cortisol (salivary cortisol correlates well with serum cortisol levels). This noninvasive method is primarily used in clinical research to study the interaction between psychological stress and the immune system (IL-1β enhances cortisol secretion, and their simultaneous measurement shows how the HPA axis is regulated). Therefore, our aim is to investigate indicators of psychological stress and IL-1β levels in patients with SD/DU, comparing their perceived stress levels and salivary cortisol and IL-1β values, and clinical features with patients with CSU and healthy controls.

## 2 Methods

### 2.1 Study design and study participants

This cross-sectional study compared data and findings of patients with SD/DU to those with CSU and healthy controls (three groups of participants), specifically, data on the intensity of perceived psychological stress (using validated questionnaires), as well as on salivary cortisol and IL-1β levels and the clinical characteristics of the disease. Furthermore, this study aimed to examine their interrelations and correlations, as well as their association with demographic data (gender, age, concomitant conditions, triggers). The purpose of the investigation was to determine whether patients with SD/DU experience higher levels of psychological stress, and whether they exhibit changed IL-1β levels compared to CSU patients and healthy controls.

A total of 100 participants were included in the study: 34 patients with SD/DU, 33 patients with CSU, and 33 healthy controls (those with SD/DU associated with CSU were excluded). The patients were triaged during routine examinations at the Allergy and Clinical Immunology Outpatient Clinic, Department of Dermatovenereology, University Hospital Center “Sestre Milosrdnice”, where the study was conducted. For all enrolled patients, disease severity and clinical parameters were evaluated. Each participant provided written informed consent, and the study received approval from the Ethics Committee of the abovementioned hospital (July 2023; number of protocol: 251-29-11-23-07). Inclusion criteria for the SD/DU and CSU groups were age ≥ 18 years; presence of wheals and/or angioedema persisting for more than 6 weeks, in accordance with current guidelines for the diagnosis of chronic urticaria (2). Patients with isolated angioedema were excluded. Exclusion criteria for all participants included use of systemic corticosteroids, antineoplastic agents, psychoactive medications, immunosuppressive/immunomodulatory drugs, or vaccines within one month prior to inclusion; use of oral contraceptives; for women, being in the follicular phase of the menstrual cycle; use of topical corticosteroids within 7 days prior to sampling; presence of systemic inflammatory or autoimmune disease, history of malignancy, diagnosed psychiatric disorder, or oral diseases; and smoking (the known influence of nicotine on salivary cortisol levels)! (24). Healthy controls were matched by age and sex to the patient groups and met the following criteria: age over 18 years, absence of dermatological, inflammatory, or autoimmune disease. For all participants, saliva samples were collected and analyzed for IL-1β and cortisol levels using the same standardized procedure.

### 2.2 Clinical assessment and questionnaires

Clinical disease parameters were evaluated using validated instruments: Urticaria Control Test (UCT) for evaluating disease control (25); Urticaria Activity Score (UAS7) for assessing disease activity in CSU patients (2); and the Perceived Stress Scale (PSS) for measuring the intensity of psychological stress (it measures stress during the period of the last 1 month) (26, 27). In addition, demographic and clinical data were collected, including age, sex, and disease duration. Further data were also analyzed, regarding comorbidities (none; allergic rhinosinusitis, asthma, pollinosis, oral allergy syndrome, concomitant type I allergies, etc.); and self-reported identified triggers.

### 2.3 Examination of wheal responses to provocation with FricTest in patients with SD/DU (dermographism activity)

**Figure 1:**
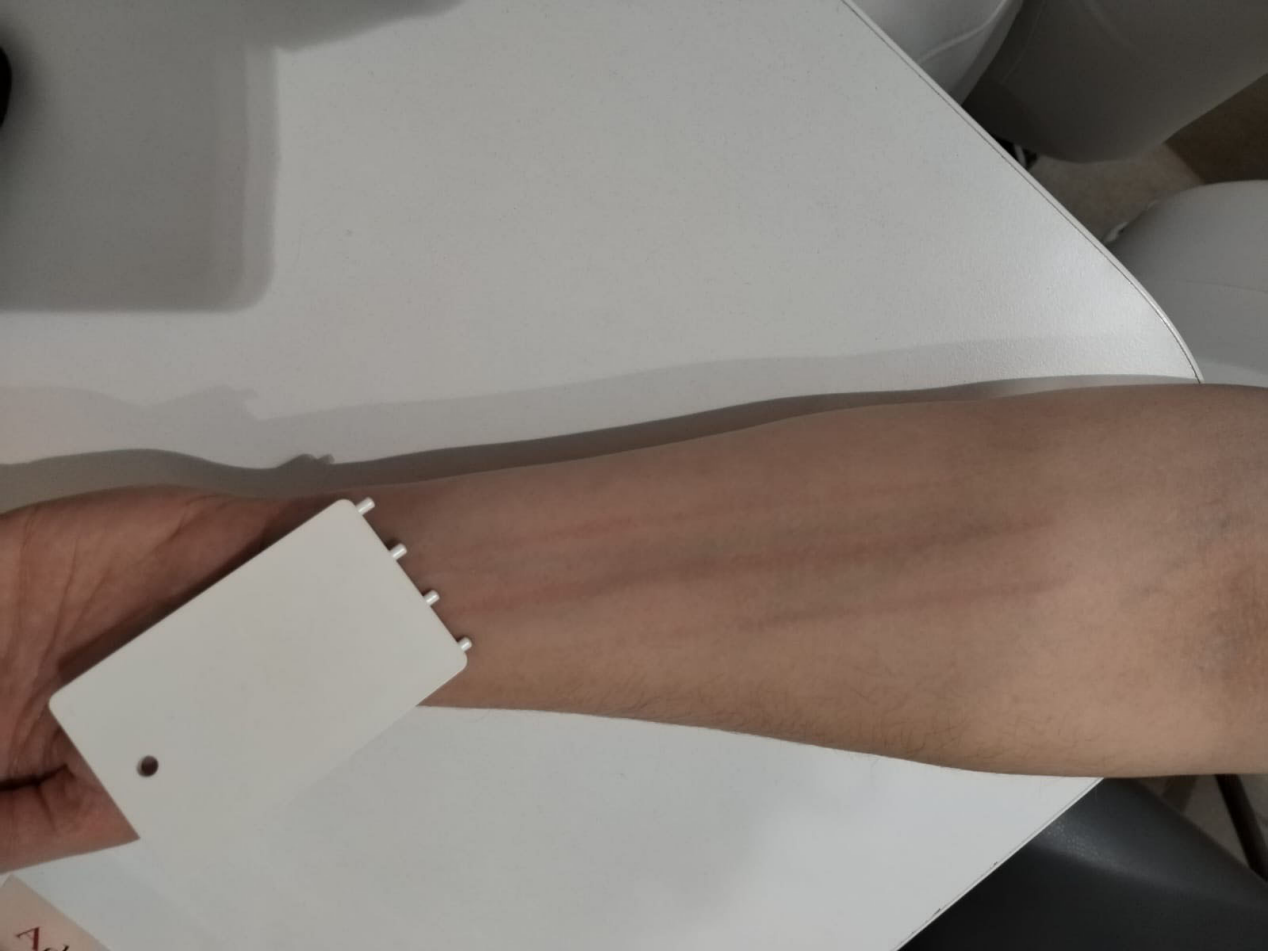
FricTest for assessing dermographism in a patient with SD/DU (photo taken with the patient’s consent).

In patients with SD/DU, we used the FricTest to assess the current severity of the dermographism, i.e., the skin’s reaction as an indicator of response to provocation testing (FricTest^®^ 4.0, Moxie, Germany), which was applied on the volar forearm of the patients (28–30) (**Figure 1**). The test was performed while patients were at rest, not taking any additional medication for urticaria, and having discontinued second-generation AHs for at least 3 days prior to testing. All procedures were conducted by the same investigator, at the same time of day between 07:00 and 09:00 a.m. We measured the wheal diameter that appeared 10 minutes after provocation. The observed responses in each patient were recorded as negative (−) or positive, scored from + to ++++, i.e. from 1–4 according to the threshold value that elicited the reaction (wheal and/or itching). Thus, we recorded the results for each patient: the higher the FricTest score, the greater the activity of SD/DU (29).

### 2.4 Saliva collection and the analysis of cortisol and IL-1β

Morning saliva samples were collected between 07:00 and 09:00 a.m., corresponding to the circadian peak of cortisol, and were used for the determination of cortisol and IL-1β concentrations (following completion of the PSS questionnaire) (26, 31, 32). Saliva collection was performed by passive drool, avoiding sampling within 90 minutes after a major meal or tooth brushing, and 12 hours after alcohol consumption. To minimize the effects of low pH and bacterial growth, participants rinsed their mouths with water 10 minutes prior to collection. They then allowed saliva to accumulate in the mouth for approximately 20 minutes, and subsequently tilted their heads forward to guide the pooled saliva into a collection vial (Salivette^®^, Sarstedt, Nümbrecht, Germany), yielding approximately 2.0–2.5 mL of saliva. Samples were transported immediately after collection to the Department of Clinical Chemistry and frozen at −20 °C within 4 hours. On the day of cortisol and IL-1β determination by Salivary ELISA Kits (Salimetrics^®^, PA, USA). The samples were thawed, vortexed, and centrifuged at 1500 x g for 15 minutes.

### 2.5 Statistical analysis

The normality of data distribution was tested using the Kolmogorov–Smirnov test. Non-normally distributed data were log-transformed to approximate normality. For normally distributed variables, comparisons among the three groups were performed using analysis of variance with the Student–Newman–Keuls *post hoc* test. For non-normally distributed variables, the Kruskal–Wallis test was applied, followed by the Mann–Whitney U test with Bonferroni correction. Effect size was calculated using the formula r = Z/√N for the Mann–Whitney U test. The correlation between variables was analyzed using Spearman’s rank correlation coefficient. The magnitude of effect size and correlation strength was interpreted according to Cohen’s criteria where *r = 0.25–0.3* indicates a small effect/low correlation, *r = 0.3–0.5* is moderate, *r = 0.5–0.7* is large, and r *> 0.7* = a very large correlation. Frequencies were compared using Fisher’s exact test. All analyses were performed using IBM SPSS Statistics, version 22.0 (IBM Corp., Armonk, NY, USA).

## 3 Results

### 3.1 Salivary cortisol and IL-1β levels, perceived stress, and clinical characteristics in patients with SD/DU, CSU, and healthy controls

**Figure 2:**
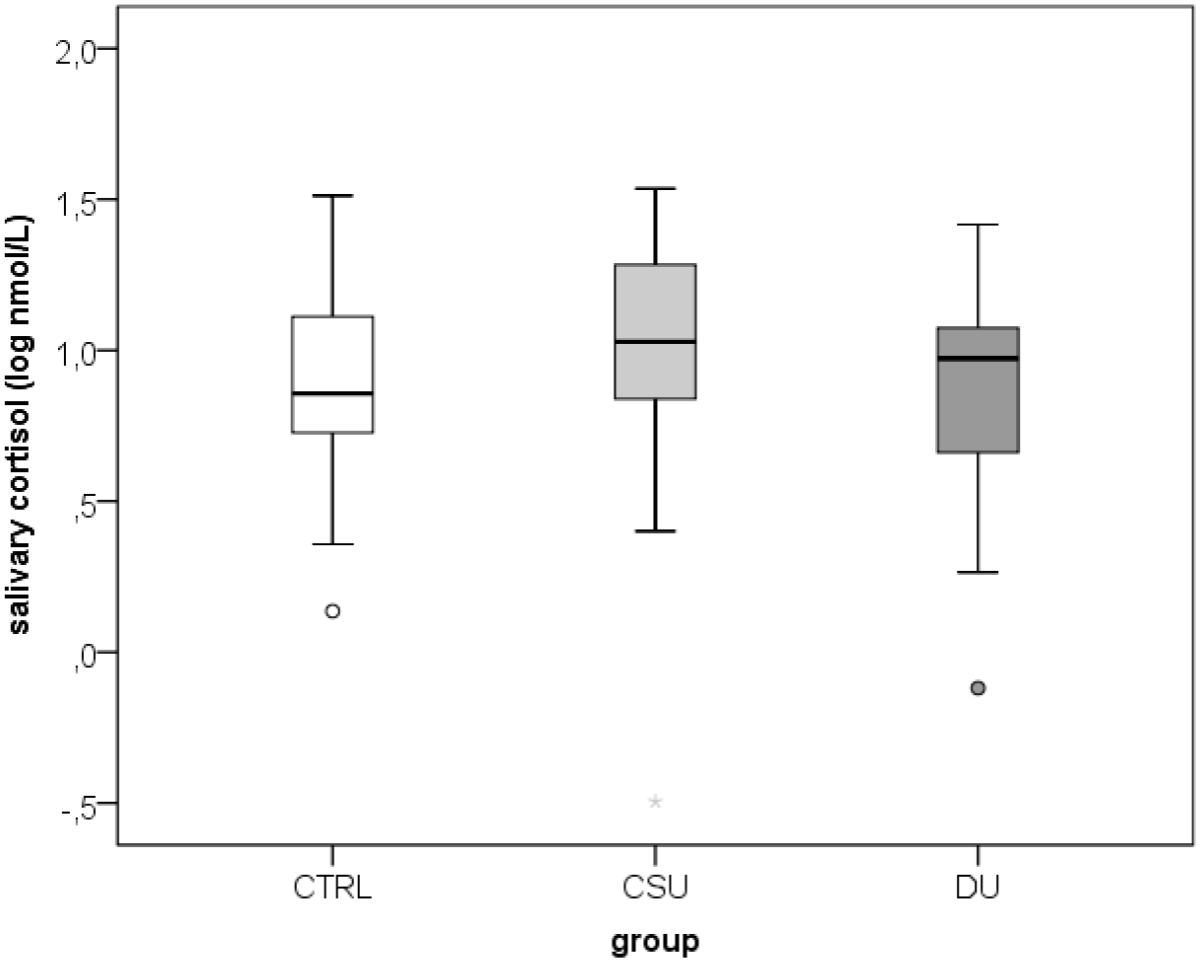
Salivary cortisol values in healthy controls and CSU patients compared to SD/DU patients. CTRL, controls; CSU, chronic spontaneous urticaria; DU, dernographic urticarial/symptomatic dermographism.

**Figure 3:**
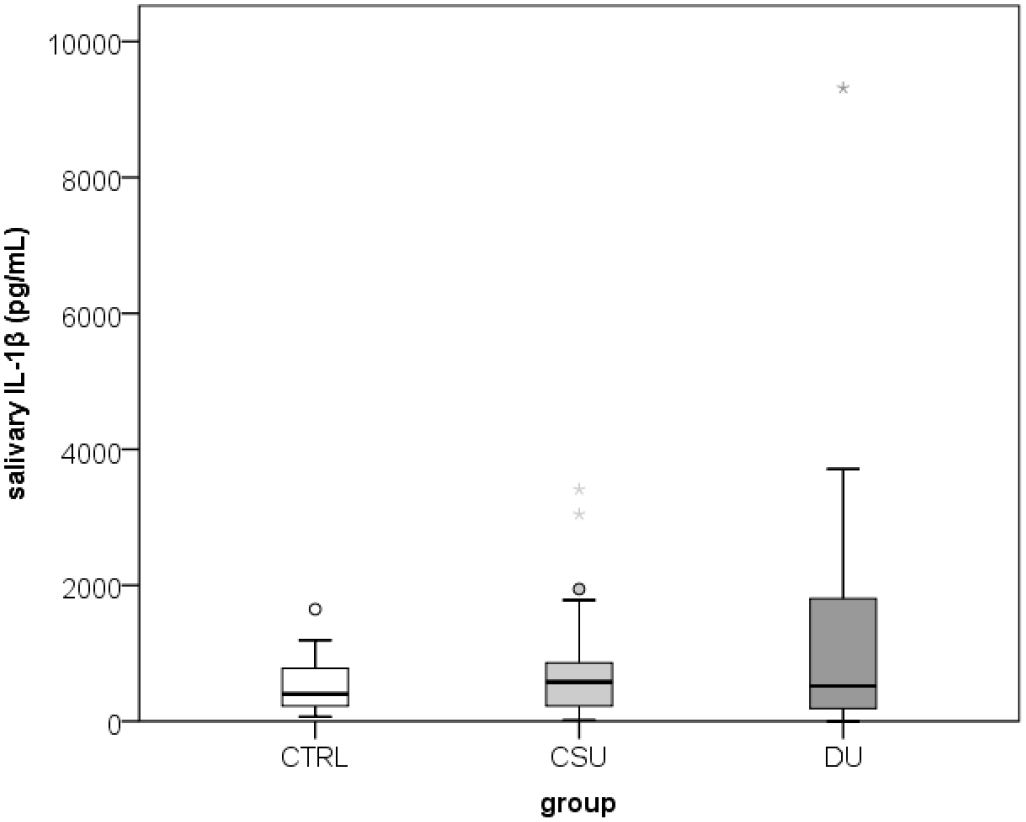
Salivary IL-1β values in healthy controls and CSU patients compared to SD/DU patients.

**Figure 4:**
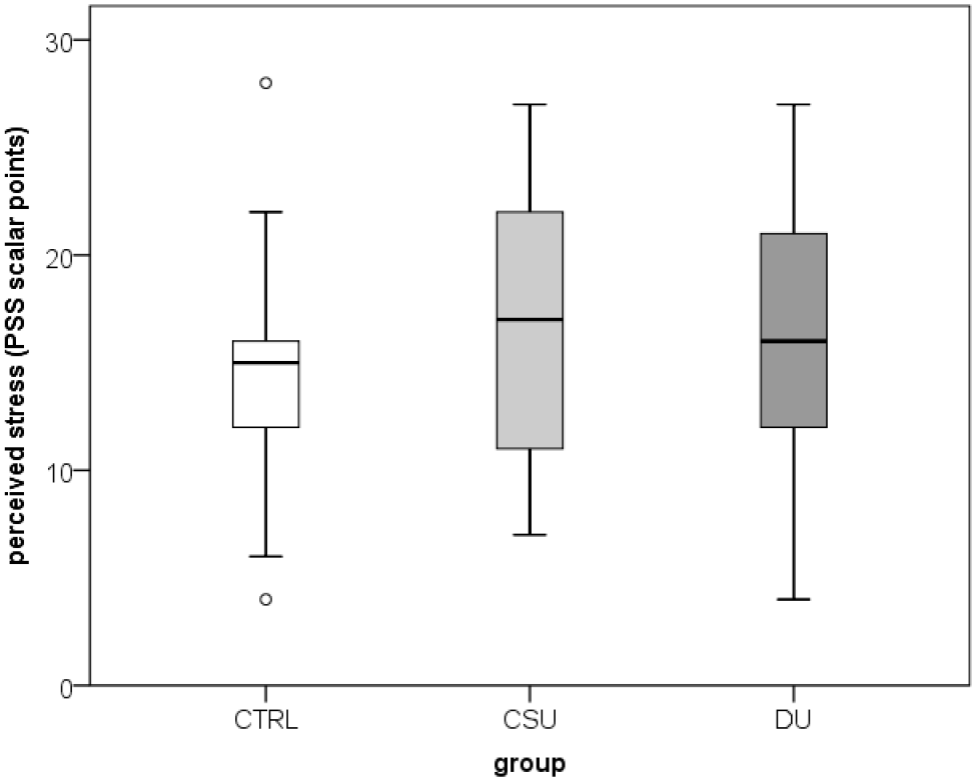
Perceived stress indicator (PSS) values in healthy controls and CSU patients compared to SD/DU patients.

**Figure 5:**
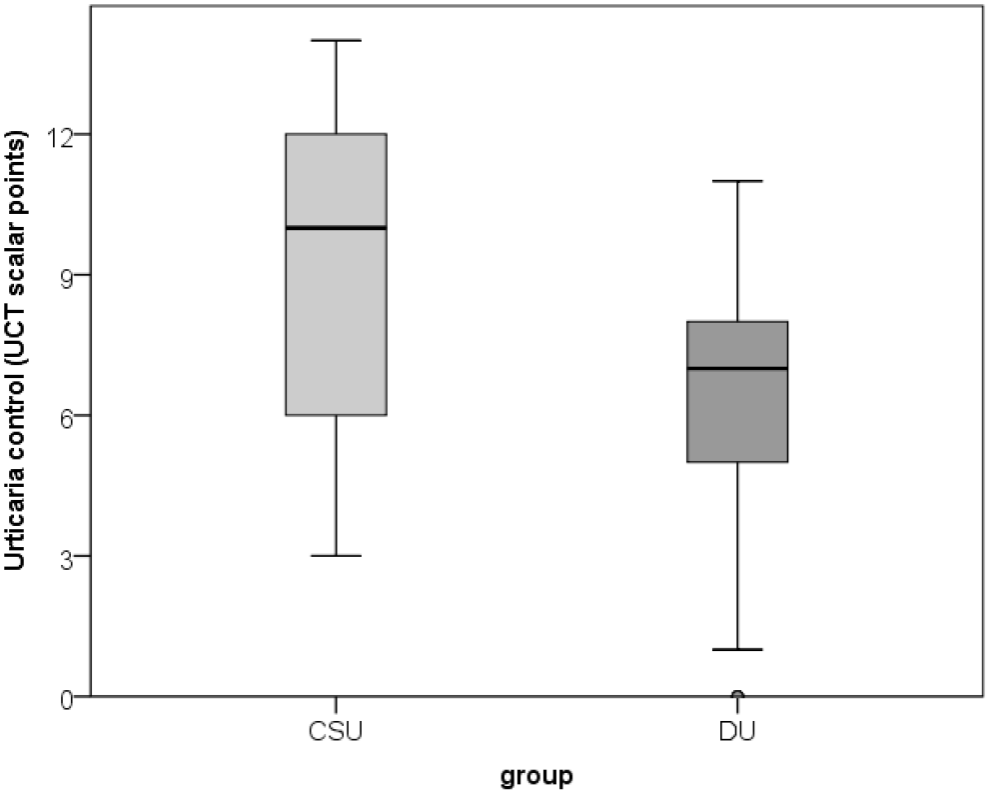
Values for urticaria control (UCT) in patients with CSU compared to SD/DU patients.

A total of 100 participants were included in the study: 34 patients with SD/DU, 33 patients with CSU, and 33 healthy controls, aged 19–64 years (median 38; interquartile range 31–48), of whom 78% were women. Age and sex distribution did not differ significantly between the three groups. Duration of SD/DU varied between 9 and 180 months, with median 36 (interquartile range 80-60). An analysis of salivary factors and perceived stress levels showed that there were no significant differences in salivary cortisol and IL-1β concentrations, or perceived stress levels (PSS scores) between the groups (**Figures 2**–**4****;****Table 1****).** When assessing disease control, the CSU group demonstrated significantly higher disease control compared to the SD/DU group, with a moderate effect size (*p = 0.001; r = 0.393*) (**Figure 5**). Disease duration in SD/DU patients was negatively linearly related to salivary cortisol (*r=-0.369; p=0.032*), but not to IL-1β. No significant correlations were observed between salivary cortisol, IL-1β, perceived stress, and age in the overall sample (including both patients and controls). Values of salivary cortisol, IL-1β, and perceived stress were not associated with patient age but did show sex-related differences, with small effect sizes. Higher IL-1β concentrations were observed in male patients compared to female patients (median 687.6 vs. 408.9 pg/mL; *p=0.029; r=0.218*). Similarly, salivary cortisol levels (log-transformed values) were higher in males than in females (median 1.1 vs. 0.9; *p=0.028; r=0.220*). Conversely, perceived stress was higher in female patients compared to males (median 16 vs. 12; *p = 0.006; r = 0.273*). For CSU patients, a significant positive correlation was found between disease severity (UAS7) and PSS (moderate, linear and positive correlation, indicating that higher perceived stress/PSS was associated with greater disease severity/UAS7). Regarding disease control and activity of two patient groups in relation to sex and age, no significant associations were found—both UCT (both diseases) and UAS7 results (CSU) were independent of sex and age.

**Table 1:** Correlation between clinical and psychoneuroimmunological parameters.

Examined factors	r and p values	IL-1β	Cortisol log	PSS	UCT	Age
IL-1β	r	1	-0.011	-0.017	0.125	-0.102
	p	.	0.931	0.894	0.315	0.409
Cortisol log	r	-0.011	1	0.019	0.181	-0.046
	p	0.931	.	0.881	0.142	0.71
PSS	r	-0.017	0.019	1	-0.156	0.098
	p	0.894	0.881	.	0.208	0.43
UCT	r	0.125	0.181	-0.156	1	0.111
	p	0.315	0.142	0.208	.	0.371
Age	r	-0.102	-0.046	0.098	0.111	1
	p	0.409	0.71	0.43	0.371	.

(IL-1β-interleukin; PSS- Perceived Stress Scale; UCT- Urticaria Control Test).

### 3.2 Analysis of additional factors in patients with SD/DU compared to patients with CSU and healthy controls

**Figure 6:**
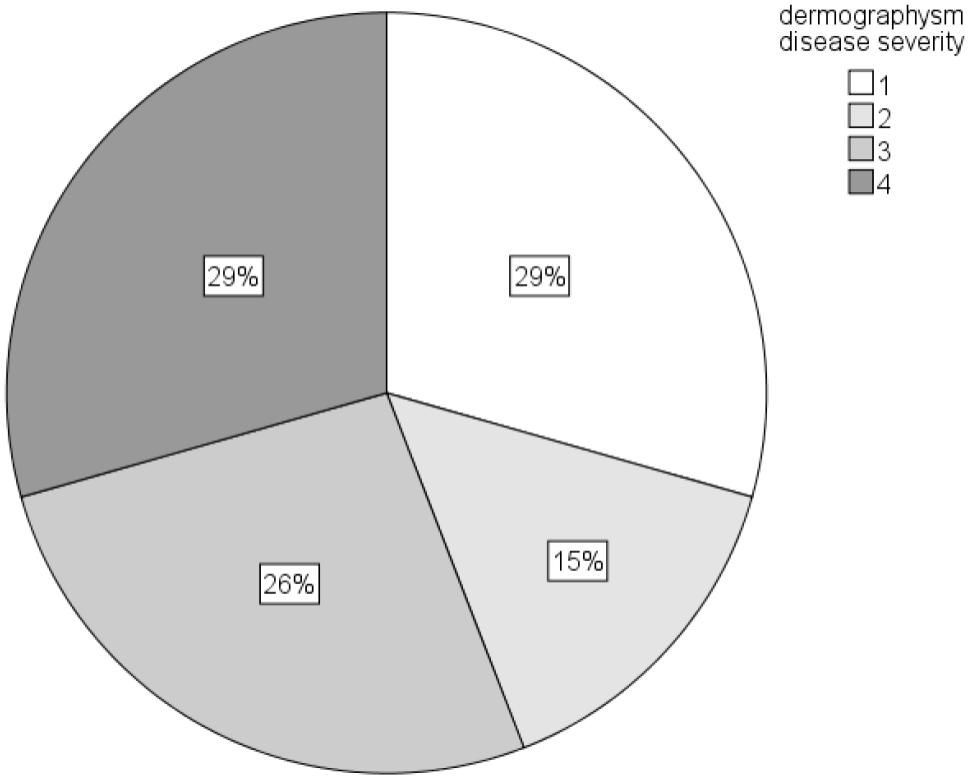
Distribution of dermographism activity/severity in patients with SD/DU (based on the test of dermographism results and the levels of dermographism activities, on scales 1-4).

**Figure 7:**
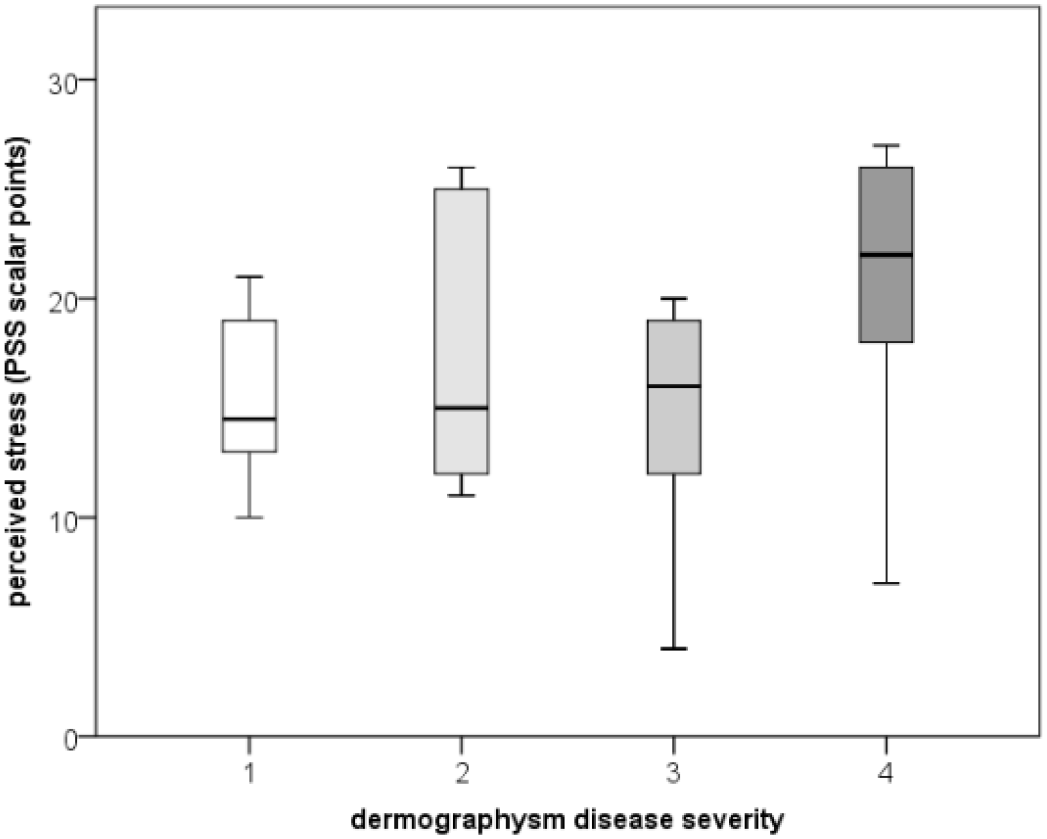
Comparison of stress levels (PSS) between dermographism activity/severity levels in patients with SD/DU.

While dermographism activity/severity (on a scale from 1 to 4) did not correlate with most examined factors (UCT, cortisol, or IL-1), they did correlate with PSS (r=0.347; p=0.045), with a positive, linear, and moderate correlation (**Figure 6****).** As perceived stress increased, so did dermographism activity levels. However, PSS did not differ significantly between dermographism activity levels (**Figure 7**). **Table 2** shows a comparison of data on concomitant/associated diseases and reported triggers for urticaria between the patient groups. SD/DSU patients who reported stress as a trigger had significantly higher disease control/UCT (8.5 vs. 6*; p=0.042; r=0.348).* Also, CSU patients who reported stress as a trigger had a higher disease severity/UAS7 (median 24 vs. 10.5; p=0.035; r=-0.362, a moderate effect size). Also, SD/DU patients with concomitant allergies had lower cortisol, with a moderate effect size (median log 1.03 vs. 0.80 nmol/L; p=0.032; r=-0.368). SD/DU lasted longer in those with concomitant conditions (60 vs. 24 months; p=0.017; r=-0.410), particularly in those with concomitant allergies (median 48 vs. 18 months; p=0.002; r=-0.51; a large effect size), especially when allergic to aeroallergens (median 54 vs. 24 months; p=0.011; r=-0.485; a moderate effect size).

**Table 2:** Comparison of concomitant/associated diseases and reported triggers for urticaria between the patient groups.

Variable	Finding	Group		p	V
		CSU (N = 33)	SD/DU (N = 34)		
Concomitant conditions	No; N (%)	25 (76%)	21 (62%)		
	Yes; N (%)	8 (24%)	13 (38%)	0.294	0.166
-allergic rhinosinusitis or asthma	Absent; N (%)	25 (76%)	23 (68%)		
	Present; N (%)	8 (24%)	11 (32%)	0.59	0.09
-oral allergy syndrome	Absent; N (%)	30 (91%)	32 (94%)		
	Present; N (%)	3 (9%)	2 (6%)	0.273	0.061
-photosensitivity	Absent N (%)	33 (100%)	33 (97%)		
	Present; N (%)	0	1 (3%)	1.000	0.121
Self-reported triggers for urticaria	No; N (%)	16 (49%)	11 (32%)		
	Yes; N (%)	17 (52%)	23 (68%)	0.218	0.164
-stress as a trigger	No; N (%)	28 (85%)	30 (88%)		
	Yes; N (%)	5 (15%)	4 (12%)	0.734	0.05
-infection as a trigger	Ne; N (%)	32 (97%)	33 (97%)		
	Yes; N (%)	1 (3%)	1 (3%)	1	0.003
-vaccination as a trigger	No; N (%)	30 (91%)	32 (94%)		
	Yes; N (%)	3 (9%)	2 (6%)	0.673	0.061
-drugs as triggers	No; N (%)	29 (88%)	28 (82%)		
	Yes; N (%)	4 (12%)	6 (18%)	0.734	0.078
-food as a trigger	No; N (%)	30 (91%)	31 (91%)		
	Yes; N (%)	3 (9%)	3 (9%)	1	0.005
-external factors as triggers	No; N (%)	28 (85%)	23 (68%)		
	Yes; N (%)	5 (15%)	11 (32%)	0.152	0.202

p-significance based on Fisher test, V-effect size.

## 4 Discussion

In this study, we focused on salivary biomarkers in SD/DU patients, given the non-invasive nature of saliva collection and the sensitivity of their skin. Salivary cortisol, in particular, provides a well-established and reliable measure of stress. While previous studies have mentioned higher stress levels in SD/DU patients, they did not measure it (33, 34). Although we did not find significant changes in salivary cortisol and IL-1β levels in our SD/DU patients, we revealed that SD/DU duration was negatively linearly related to salivary cortisol (*r=-0.369; p=0.032*). According to literature data, while acute/short-term cortisol release supports the control of acute inflammation, chronic stress leads to sustained high cortisol levels (decreased cortisol secretion), which can suppress immune function and IL-1β production, increasing susceptibility to chronic inflammation (13, 35). Our patients perceived high stress levels, but their cortisol levels were not high but low, which is in accordance with literature data that long-lasting stress may decrease morning cortisol levels due to exhausted HPA axis (13). Also, our results in men revealed a significant correlation between dermographism activity and perceived stress/PSS and higher cortisol and IL-1β values than women, although women reported higher perceived stress/PSS. However, literature data on SD/DU features is scarce, the majority are retrospective studies or based on questionnaires (36–38). Among our SD/DU patients, women and those between 30 and 40 years old predominated, similarly to Slovenian retrospective study (on SD/DU and CSU patients) which found predominance of women (65% in SD/DU, 66% in CSU), where SD/DU patients were significantly younger (median: 37 years) and had earlier disease onset (median: 34 years), longer duration (12 *versus* 6 months), and less frequently relapsed than CSU (1.0% vs. 16.7%) (37). In one internet-based research on SD/DU (Thailand), females also predominated (with ratios of 2.2-2.4:1), disease onset was 16 years; higher probability of SD/DU was seen in atopics (with allergic conjunctivitis and atopic dermatitis) and those with older age (38). According to one retrospective study (Turkey), SD/DU patients had shorter disease duration (median 12 months) (in our study it was 36 months) and lower AH use, but significantly more commonly were atopics (asthma, allergic rhinitis) than CSU patients (39). Similarly, about one third of our SD/DU patients had concomitant atopic diseases, supporting previous study results. Regarding treatment options other than AHs, for instance for patients with chronic stress-related urticaria, stress management measures, such as relaxation, mindfulness or cognitive behavioral therapy, could be tried.

### 4.1 Limitations and future directions

The limitations of this study (which was the first simultaneously analyzed psychological stress, immunological factors and clinical features in SD/DU patients) are the relatively small number of patients and the lack of other potentially valuable data like stress duration (considering medical data that long-lasting stress can reduce morning cortisol level), as well as the possibility that salivary IL-1β is a potentially more useful indicator of oral conditions, and not reliable indicator of chronic urticaria. Since our results indicate high perceived stress in SD/DU patients and since the duration of their illness was negatively linearly related to salivary cortisol (supporting chronic stress), reducing psychological stress could help improve the condition of SD/DU. This highlights the need for further studies of biomarkers of SD/DU and associated stress and for larger, more comprehensive studies.

## Data Availability

The original contributions presented in the study are included in the article/supplementary material. Further inquiries can be directed to the corresponding author.
